# Nintedanib regulates intestinal smooth muscle hyperplasia and phenotype in vitro and in TNBS colitis in vivo

**DOI:** 10.1038/s41598-022-14491-5

**Published:** 2022-06-17

**Authors:** Jay Kataria, Jack Kerr, Sandra R. Lourenssen, Michael G. Blennerhassett

**Affiliations:** Gastrointestinal Diseases Research Unit, Department of Medicine, GIDRU Wing, Kingston General Hospital, Queen’s University, Kingston, ON K7L 2V7 Canada

**Keywords:** Cell biology, Gastroenterology, Medical research, Pathogenesis

## Abstract

Chronic inflammation of the human intestine in Crohn’s disease (CD) causes bowel wall thickening, which typically progresses to stricturing and a recurrent need for surgery. Current therapies have limited success and CD remains idiopathic and incurable. Recent evidence shows a key role of intestinal smooth muscle cell (ISMC) hyperplasia in stricturing, which is not targeted by current anti-inflammatory therapeutics. However, progression of idiopathic pulmonary fibrosis, resembling CD in pathophysiology, is controlled by the tyrosine kinase inhibitors nintedanib (NIN) or pirfenidone, and we investigated these drugs for their effect on ISMC. In a culture model of rat ISMC, NIN inhibited serum- and PDGF-BB-stimulated growth and cell migration, and promoted the differentiated phenotype, while increasing secreted collagen. NIN did not affect signaling through PDGF-Rβ or NFκB but did inhibit cytokine-induced expression of the pro-inflammatory cytokines IL-1β and TNFα, supporting a transcriptional level of control. In TNBS-induced colitis in mice, which resembles CD, NIN decreased ISMC hyperplasia as well as expression of TNFα and IL-1β, without effect in control animals. NIN also inhibited growth of human ISMC in response to human serum or PDGF-BB, which further establishes a broad range of actions of NIN that support further trial in human IBD.

## Introduction

### Crohn’s disease

The chronic intestinal inflammation of Inflammatory Bowel Disease (IBD) affects an increasing number of people, particularly in the developed world, with significant health and economic consequences. For example, the prevalence of IBD was 1.3% in the United States in 2015 and afflicted nearly 1% of Canadians in 2021^1,2^. Crohn’s disease (CD) is the subtype of IBD that causes transmural segmental inflammation of the intestine, with a predilection for the terminal ileum. Stricturing disease in CD is common, where the remitting and relapsing pattern of inflammation causes a thickened, noncompliant and obstructive region of intestine that requires surgical excision, but commonly recurs. Already present in 20% of patients at diagnosis, this typically affects more than 50% of CD patients over time^3–5^.

Currently, the therapies for CD are broadly anti-inflammatory, such as immunomodulators and anti-TNFα immunotherapies, and directed largely against immune cells^5^. However, these have only limited effects on stricturing, typically decreasing or slowing stricture formation without preventing occurrence and most patients still progress to require surgery; the other newer biological therapies have not yet shown effect against stricturing disease^6^. This emphasizes the need for identification of cellular mechanisms that contribute to stricture formation as well as the ability to selectively target these processes in the inflamed region (reviewed^7^).

### The pathophysiology of stricture formation is ill defined

The cause of stricture formation is unclear. There is clearly an interaction between an activated immune system and the local stromal cells that over time drives the progression of fibrosis, as occurs elsewhere (e.g., the respiratory and vascular systems), since there is increased extracellular matrix (ECM) that affects tissue function and contributes to altered compliance^8^. However, the principal component of obstructive stricturing is the increased smooth muscle mass, arising from hypertrophy of the muscularis propria along with migration and hyperplasia of the intestinal smooth muscle cells (ISMC)^3,9^. The smooth muscle cell is thus a key cellular contributor to these outcomes.

Some understanding of the effects of inflammation on the ISMC and their subsequent responses has been derived from trinitrobenzene sulfonic acid (TNBS)-induced colitis in rodents. A widely used model of transmural disease that resembles aspects of CD, this has a cellular mechanism of regional hyperplasia and hypertrophy of the ISMC with the appearance of an altered phenotype, as well as sporadic progression to stricture formation^10–13^.

Overall, this model aligns with human pathology in identifying ISMC hyperplasia and altered collagen production as key features of persistent regional inflammation that correlate with stricture formation in both humans and animal models^9,12^.

### Phenotypic modulation of the smooth muscle cell

The mature smooth muscle cell retains the ability to dedifferentiate or modulate its phenotype to undergo cell division as a normal response to injury. However, ongoing inflammation may contribute to continued proliferation that is associated with pathology in the cardiovascular system and elsewhere. In contrast to the transient growth and normal restoration of phenotype, protracted stimulation of growth increases smooth muscle mass but also leads to progressive de-differentiation and a reduced ability to re-differentiate (reviewed^14^). In the intestine, recent work shows that protracted growth of rat ISMC in vitro caused epigenetic changes responsible for decreased expression of phenotypic markers typical of rat intestinal strictures, and indeed, already present in human CD strictures^12,15^.

The major growth stimulus for ISMC is platelet-derived growth factor-BB (PDGF-BB), acting via its receptor PDGF-Rβ following its induction by inflammatory cytokines^16,17^. Elsewhere, PDGF-BB also triggers altered gene expression that compromises the contractile phenotype of smooth muscle cells^18,19^, adding to the case for PDGF-BB as a major participant in the hyperplasia and altered phenotype of ISMC in Crohn’s strictures.

### IPF: chronic inflammation of the airway

While the events that lead to stricture formation remain ill-defined, integration with study of other disease processes with common elements can provide new avenues for research. Idiopathic pulmonary fibrosis (IPF) is a chronic, progressive disease of airway inflammation with loss of lung function that, like CD, is hypothesized to result from an altered repair process and abnormal tissue remodeling^20^. There is alveolar epithelial cell injury and expansion of mesenchymal cells, with increased appearance of myofibroblasts^21^ and expansion of the airway smooth muscle^22,23^, along with activation of tyrosine kinase-dependent signaling pathways such as VEGF, FGF, and PDGF^24^. Activation of these pathways is associated with growth stimulation and expansion of the mesenchymal compartment as well as diverse aspects of the immune response^20^. While IPF has been refractory to therapy, nintedanib (NIN; BIBF 1120) and pirfenidone have been found to achieve control of disease progression^25^, suggesting a similar approach may apply to CD. These novel, multi-modal agents result in disease control, with reduced rate of decline in forced vital capacity, fewer acute exacerbations, and increased health-related quality of life^24^. Their mechanism of action is ill-defined, with nintedanib described as an inhibitor of multiple tyrosine kinases in endothelial cells, pericytes, and smooth muscle cells.

We proposed that these drugs could exert a similar effect in CD and explored this using established models of circular smooth muscle cells (CSMC) from the rat colon in vitro, as well as the TNBS model of colitis in vivo. Early results showed NIN to be a potent and selective inhibitor of CSMC proliferation, more so than pirfenidone, and we pursued the effects of NIN on the maintenance of CSMC phenotype and the expression of extracellular matrix, as well as its impact on cytokine-induced expression of pro-inflammatory cytokines by CSMC. Combined with pilot work showing beneficial outcomes in TNBS-colitis in mice and in initial work on human CSMC, our work suggests that NIN may be able to target the role of smooth muscle in the pathophysiological processes that cause stricture formation in CD.

## Methods

### Animals and colitis

Sprague–Dawley male rats and BALB/c male mice were obtained from Charles River Laboratories (Montreal, QC) and housed in microfilter isolated cages with free access to water and food. This study was carried out in compliance with the ARRIVE guidelines, and all procedures were approved by the Queen’s University Animal Care Committee and adhered to the Canadian Council of Animal Care’s policies. All methods were performed in accordance with the relevant guidelines and regulations.

Colitis was induced in the mouse by instillation of 100 μL of 5% (w/v) TNBS (MilliporeSigma) dissolved in 50% ethanol into the colon 4 cm proximal to the anus. Nintedanib (NIN; 0.5 mg/200 µL per mouse, LC Labs, MA, USA) was dissolved in water and administered by gavage one day before the induction of TNBS colitis, then daily before sacrifice at 2 or 6 days post-induction of colitis. Control animals received either NIN or water. At specified times, animals were sacrificed by cervical dislocation under isoflurane anesthesia and colons were removed and processed for histology, immunocytochemistry, or qPCR.

### Tissue culture

#### Isolation of rat circular smooth muscle

The colon of adult male rats was dissected, opened, and pinned to a Sylgard-coated plate. Strips of circular muscle were removed with microdissection, then dissociated in HEPES-buffered digestion solution [papain (0.5 mg/mL), BSA (1 mg/mL), dithiothreitol (1 µM), and collagenase (type F, 0.25 mg/mL)] as described previously^15^. These and all other reagents are from Millipore Sigma, Ontario, Canada unless specified. Cells were then incubated at 4 °C for 2 h, at room temperature for 1 h, and then at 37 °C for 1 h, followed by trituration and plating onto 60-mm culture dishes in DMEM containing 5% fetal calf serum (FCS). Culture purity was confirmed by the lack of immunostaining for glia or interstitial cells of Cajal, as described previously^17^.

Once cultures reached confluence, they were passaged (1:2 ratio) ≥ 10 times to generate high-passage cells. In some experiments, cells were passaged onto collagen-coated glass cover slips in 24-well plates for immunocytochemistry or collagen staining, as described below. These plates were also used without added coverslips to obtain cells for subsequent western blot analysis or qPCR. Alternatively, cells were plated into wells of 96 well plates for growth assays. For experiments involving collagen detection, ascorbic acid-2-phosphate (0.05 mM) was included in the incubation medium.

Each cell line (“n”) was derived from a distinct animal and their use is reported with each “n” representing the averaged outcome of replicates from a single cell line.

#### Isolation of human circular smooth muscle

Human tissue was obtained in accordance with relevant guidelines/regulations, and informed consent was obtained from all participants and/or their legal guardians. Following approval from the Health Sciences Research Ethics Board of Queen’s University (file no. PATH-151-15), human tissue from the terminal ileum was obtained following resection for colon cancer. Processing to obtain cultures of circular ileal smooth muscle was described earlier^15^. Briefly, strips of circular smooth muscle were dissected and dissociated as described for rat tissue, except the first incubation was at 4 °C overnight.

Each human cell line was derived from a distinct human, and outcomes for a cell line are reported as the average of replicates; “n” thus represents the number of distinct cell lines used.

#### Treatment of cell cultures

Rat or human smooth muscle cells were maintained similarly, in DMEM with 5% FCS. To assess cell viability and growth responses, cells were plated at equal cell number [ca 3000 (rat) or 2000 (human) cells/well] in a 96-well plate, incubated overnight before washing and treatment with serum [FCS 5%, rat (RS) or human serum (HS) 2.5%] or growth factors in DMEM. NIN (0.001–800 μM) or pirfenidone (0.01–8 mM) was added 30 min prior to the addition of PDGF-BB (50 ng/mL; Peprotech, NJ, USA) and incubated at 37 °C for 48 h, followed by assay for cell viability at + 6 h, or cell growth + 48 h using the wst-8 assay kit (Millipore Sigma). Specifically, wells were washed in phenol red-free DMEM (Fisher, ON, Canada) before addition of wst-8 solution diluted in phenol red-free DMEM and incubation for 1 h. Outcomes were measured at 450 nm using a Spectra Max M3 plate reader (Molecular Devices, CA, USA), with all conditions assayed in triplicate.

The rat-derived intestinal epithelial cell line IEC-18 and the rat fibroblast cell line 3T3 were a gift from MJ Ropeleski, MD (Queen’s University). These were maintained in DMEM with 5% FCS and assayed as confluent monolayers.

### Western Blot Analysis

Smooth muscle cells were cultured in a 24-well plate at 10^5^ cells/well in 5% FCS before exposure to treatment conditions for 48 h. Cells were then scraped into equal volumes of sample buffer (Tris/HCl pH 6.8, glycerol, SDS, 2-β mercaptoethanol, bromophenol blue) and stored at − 80 °C. Samples were electrophoresed using a 12% SDS-PAGE gel, then transferred onto a PVDF membrane. This was followed by incubation in 5% milk diluted in Tris-buffered saline containing 0.1% Tween-20 (TBS-T; Bioshop, ON, Canada), then in antibodies diluted in TBS-T (anti-SMA (1:1000; mouse; Novus, ON, Canada) and anti-SM22 (1:5000; rabbit; Abcam, ON, Canada) at 4 °C overnight. This was followed by a 2 h incubation in appropriate secondary antibodies (anti-mouse HRP; 1:20,000; Fisher, anti-rabbit HRP; 1:4000; NEB, ON, Canada). Membranes were then exposed to a chemiluminescent substrate (Luminata Forte) and imaged using a ChemiDoc MP System (Bio-Rad, ON, Canada). Images of bands exposed until just below band saturation were quantified using Image Lab software (Bio-Rad). The membrane was then reprobed with anti-GAPDH antibodies (1:5000) as above for normalization to loading volume.

### Collagen detection

#### Sircol red assay

Intracellular and extracellular collagen was detected in rat CSMC using a modified protocol^26^. Equal volumes of lysate or supernatant were mixed with 0.5 M acetic acid, treated with pepsin (1 µg/mL; 1 h) to digest non-collagen protein, and incubated with Sirius Red (0.1% in saturated picric acid) for 30 min. The solution was centrifuged at 14,000 rpm for 10 min and the supernatant was discarded. The pellet was solubilized in NaOH (0.5 M) for 10 min before measurement of absorbance values at 550 nm using the Spectra Max M3 plate reader. Outcomes were converted to collagen content by comparison with control reactions using defined amounts of acid soluble rat tail collagen with all experiments conducted in triplicate.

Coomassie Blue stain was used to confirm that the precipitate formed from the Sirius Red assay was collagen. The precipitate was dissolved in 50 μL water or 50 μL acetic acid (0.5 M). Next, 30 μL of the solution was loaded into wells of a 1.0 mm thick 6% SDS-PAGE gel. Samples were electrophoresed (200 V for 10 min., 120 V for 1 h) and the gel was incubated for 4 h in Coomassie Blue staining solution (1 g Coomassie Brilliant Blue (Bio-Rad) in 1 L of 50% methanol, 10% glacial acetic acid, and 40% water). The gel was then washed in water until bands were visible and the MW was verified as 130 kDa^27^.

#### Sirius red histochemistry

Cells cultured on coverslips were fixed for 10 min with 4% neutral buffered formalin (NBF), washed with phosphate buffered saline (PBS), then dried at 37 °C for 1 h. Cells were then incubated in Sirius Red solution (0.1% in 0.01 N HCl) for 1 h and washed with 0.01 N HCl for 10 min. Coverslips were dehydrated with increasing concentrations of ethanol and mounted on glass slides using Permount. Representative images were captured with an Infinity 2 camera (Lumenera, ON, Canada) attached to an Olympus microscope (BX60; Olympus, ON, Canada).

### Quantitative PCR analysis

RNA was extracted from cell cultures or the muscularis externa of the mouse colon using the EZNA Total RNA Kit I (OMEGA Bio-tek; GA, USA) according to the manufacturer’s instructions. An iScript cDNA Synthesis Kit (BioRad) was used to convert RNA to cDNA, and reactions were carried out using a Step One Plus real-time PCR system (Applied Biosystems, ON, Canada), cycled for 3 s at 95 °C, then 30 s at 60 °C for 40 cycles for all primers. Values were normalized to hypoxanthine–guanine phosphoribosyltransferase (HPRT) mRNA levels after confirmation of PCR amplification efficiencies as described previously (Bonafiglia). Dissociation curve analysis confirmed a single reaction product. The ΔΔCt method of relative quantification was used to determine relative changes in mRNA expression, which are presented as the average of independent outcomes. Primer sequences are listed in the Supplementary Table 1 (rat) and Supplementary Table 2 (mouse).

### Immunocytochemistry and image analysis

Immunocytochemistry was used to detect changes in expression of SMA and SM22 in high passage rat CSMC at 48 h post treatment with NIN (1 µM). Cells were fixed with NBF followed by 100% ethanol for 1 min. Cover slips were then washed with PBS, incubated in 1% goat serum for 1 h, then exposed to anti-SMA (1:500; mouse) and anti-SM22 (1:2000; rabbit) diluted in PBS containing 0.2% Tween-20 (PBS-T) overnight at 4 °C. This was followed by a 2 h incubation with Alexa-linked secondary antibodies [(488 goat-anti mouse (1:1000; Fisher), and 555 goat anti-rabbit (1:2000; Fisher)]. Hoechst 333258 (0.1 μL/mL) was added for 1 min to label all nuclei. To examine PDGF-Rβ localization, cells were stained with antibodies against PDGF-Rβ (1:250; NEB) as indicated above. To study the activation of the NFκB pathway in response to cytokine addition, CSMC were treated with TNFα (Peprotech; 0.001–50 ng/mL) for 15, 30, or 60 min, and were fixed and processed to detect p65NFκB (1:200; NEB) expression as above. Outcomes were visualized and analyzed using an Olympus BX51 microscope attached to a Retiga 2000R CCD camera (QImaging, BC, Canada) as described previously^15^.

Standardized exposure times were used to capture optimal images without saturation for analysis (Q-Capture Pro 7, QImaging). Expression levels of contractile markers in cultures were quantified by measurement of the average optical density of cytoplasmic regions of cells that intersected an arbitrary image midline, carried out by an observer blinded to the experimental conditions. Six non-adjacent cells were analyzed per image. To measure the effect of treatment of cell membrane localization of PDGF-Rβ, individual line scans (3.5 µM) across cell margins were normalized for intensity of receptor expression for 3 non-adjacent images for condition, using a 60 × water immersion objective (1.4 NA; Olympus). For analysis of p65NFκB expression, 3 non-adjacent images per condition were analyzed for the number of cells with nuclear labeling relative to the total number of nuclei in each image. All images were analyzed using ImagePro Plus 6.0 (Media Cybernetics, MD, USA).

### Scrape wounding

A scrape wounding model was used to quantify CSMC migration. For this, cells were incubated in DMEM ± PDGF-BB (50 ng/mL) or RS (2.5%). Some wells were pretreated with NIN (1 µM), Imatinib (2 µM; LC Laboratories, MA, USA), or thymidine (2 mM) to block DNA synthesis. A 200 µL pipette tip was used to scrape a cross-shaped mark on confluent cultures and photographed using a low power phase contrast objective (4×; Olympus IMT2 microscope). The fields were re-imaged 18 h later, obtaining 10 × phase contrast images at 4 sites 200 μm apart on each arm of the initial cross-shaped wound. Image analysis was used to measure the distance travelled by the advancing edge of the cell sheet and the average cell migration was then calculated. Each condition was assayed in duplicate for each of 3 different cell lines. Each overall experiment was repeated 3 times per cell line.

### Histology and immunohistochemistry

An inflammation score for colonic disease consisted of the combination of macroscopic and microscopic scores (0–10). Upon tissue retrieval, macroscopic damage was assessed on a scale of 0–5 at the time of tissue removal according to a previously established score^28^. After processing, 4 µm sections of control or inflamed colons were stained with hematoxylin and eosin for routine examination of cellular histology. Inflammation of the circular smooth muscle layer of four non-adjacent fields per cross-section was scored as reported, using a scale (0–5) where 0 represented the absence of immune cells and 5 was indicative of a circumferential immune cell infiltrate^10^.

Low magnification images were used to measure the total cross-sectional area of 3 non-adjacent images for each colon as described previously^11^. Consecutive sections were labeled with anti-SM22 antibodies and Hoechst as above, and 5–6 non-adjacent images per section were analyzed for CSMC number in a given area of circular smooth muscle to determine the number of CSMC/cross-section. Similarly, sections from colon removed at Day 2 of colitis were co-labeled with anti-SMA (1:1000) and anti-KI67 (1:100; NEB) antibodies, followed by secondary antibody labeling as above. The number of co-labeled CSMC/cross-section was measured in 2–3 non-adjacent cross-sections/colon and used to determine the percent of proliferating CSMC/cross-section.

### Statistics

Values are presented as mean ± SE of n independent cell lines, with each cell line derived from different animals or human tissues. Differences between control and treatment conditions are considered significant for p ≤ 0.05 by ANOVA or Kruskal–Wallis test with Dunnett’s post hoc test. Differences between two groups are considered significant for p ≤ 0.05 by one- or two-tailed Student’s t-test.

## Results

**Figure 1:**
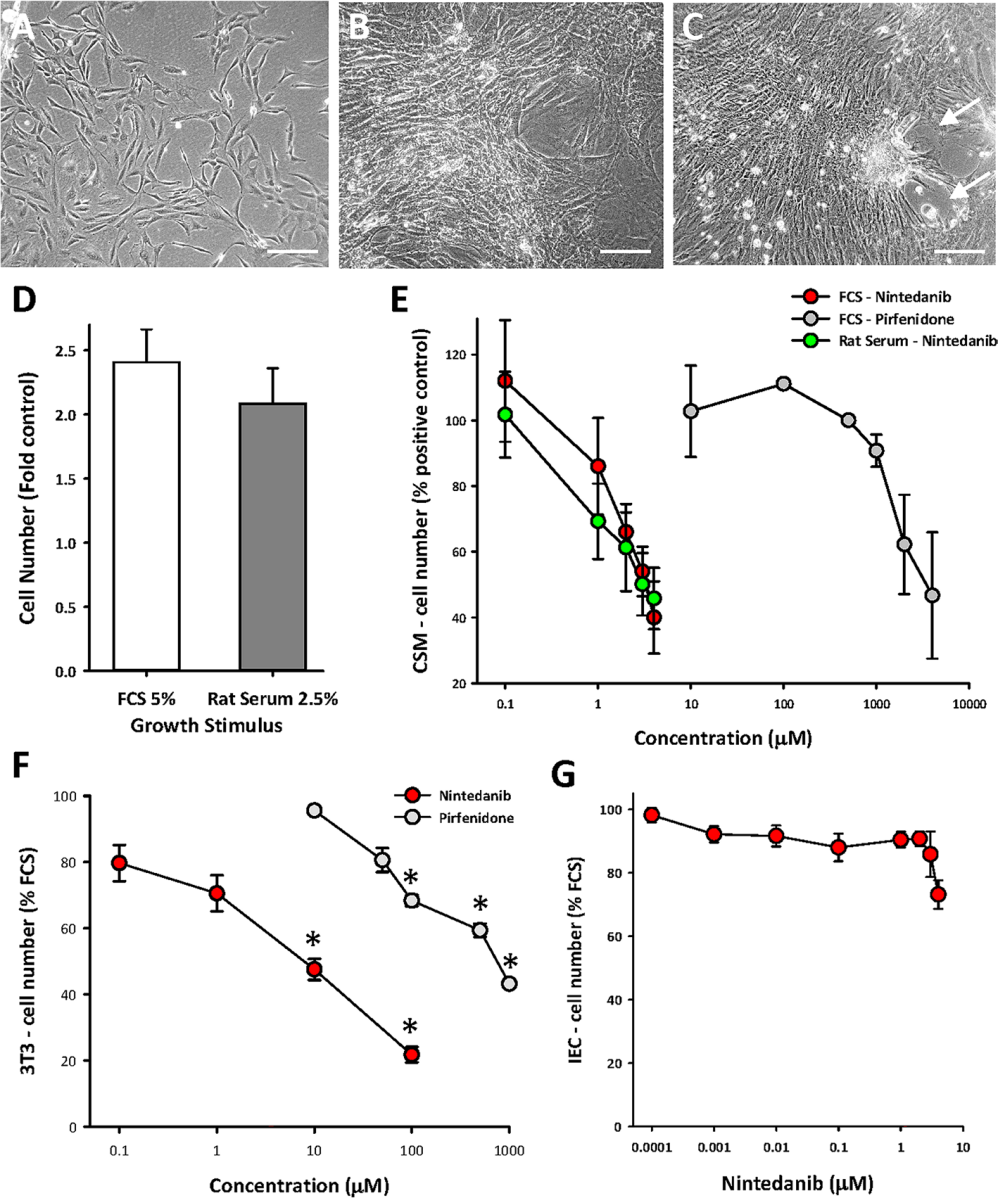
NIN limits serum-stimulated growth of smooth muscle cells from circular smooth muscle layer of adult rat colon (CSMC) in vitro. (**A**–**C**) Representative phase contrast images of culture model of CSMC at passage 14 in vitro after plating, showing sub-confluent appearance (**A**), progressive growth to post-confluence with overlapping cell layers (**B**) and retraction in local areas at when maintained at high density (e.g., arrows) (**C**). Scale bars, 200 µM. (**D**) Growth assays showing similar outcomes after 48 h stimulation between fetal calf serum (FCS, 5%) and adult rat serum (RS, 2.5%), with p < 0.05 vs control, n = 5 cell lines, each with triplicate assays. (**E**) Nintedanib inhibits stimulation of growth of CSMC by FCS or RS with greater sensitivity than pirfenidone. p < 0.05 vs control for all data with nintedanib > 0.1 µM; p < 0.05 for pirfenidone ≥ 2 mM; n = 5 cell lines with triplicate assays. (**F**) Similar outcomes to (**E**) for inhibition of FCS-induced growth of fibroblast 3T3 cells. *p < 0.05 vs control. (**G**) Lack of effect of nintedanib on FCS-induced growth of rat intestinal epithelial cells (IEC-18).

A model system of high passage rat CSMC cell lines (passages 14–20) was used as described earlier. Here, epigenetic modification of phenotype occurs with proliferation, and the CSMC resemble those obtained directly from the human stricture in CD^15^. These rat CSMC displayed the typical molecular characteristics of intestinal smooth muscle as described earlier (e.g., Refs.^12,15^) and appeared as bipolar spindle- or ribbon-shaped cells with progressive alignment as density increased. This led to overlapping layers of cells with localized areas of spontaneous retraction (Fig. 1A–C). Quantitative assays of cell proliferation expressed outcomes as the average of data from independent cell lines, with each line derived from a distinct adult rat. This showed a two- to three-fold increase in cell number over 48 h in response to fetal calf serum (FCS), and a similar outcome was seen with adult rat serum (RS), used to optimize relevance to conditions in vivo (Fig. 1D).

This assay showed that both of the novel multi-modal drugs nintedanib (NIN) and pirfenidone could inhibit FCS-induced growth of CSMC, but with markedly different abilities—NIN was roughly 1000-fold more effective than pirfenidone and displayed similar effectiveness in inhibition of FCS vs RS (Fig. 1E). These drugs have been studied in lung fibrosis, where both pulmonary smooth muscle and fibroblasts participate (e.g., Ref.^29^), and so we also examined their effect on fibroblasts, using the 3T3 fibroblast cell line. Again, FCS-induced growth of 3T3 cells showed a far greater sensitivity to NIN (Fig. 1F), so that future studies remained focused on this agent.

In contrast to its action on smooth muscle and fibroblasts, NIN was essentially ineffective against FCS-induced growth of a rat-derived intestinal epithelial cell line (IEC-18; Fig. 1G), interpreted as evidence for selectivity of NIN to inhibit proliferation of cells with mesenchymal origin.

**Figure 2:**
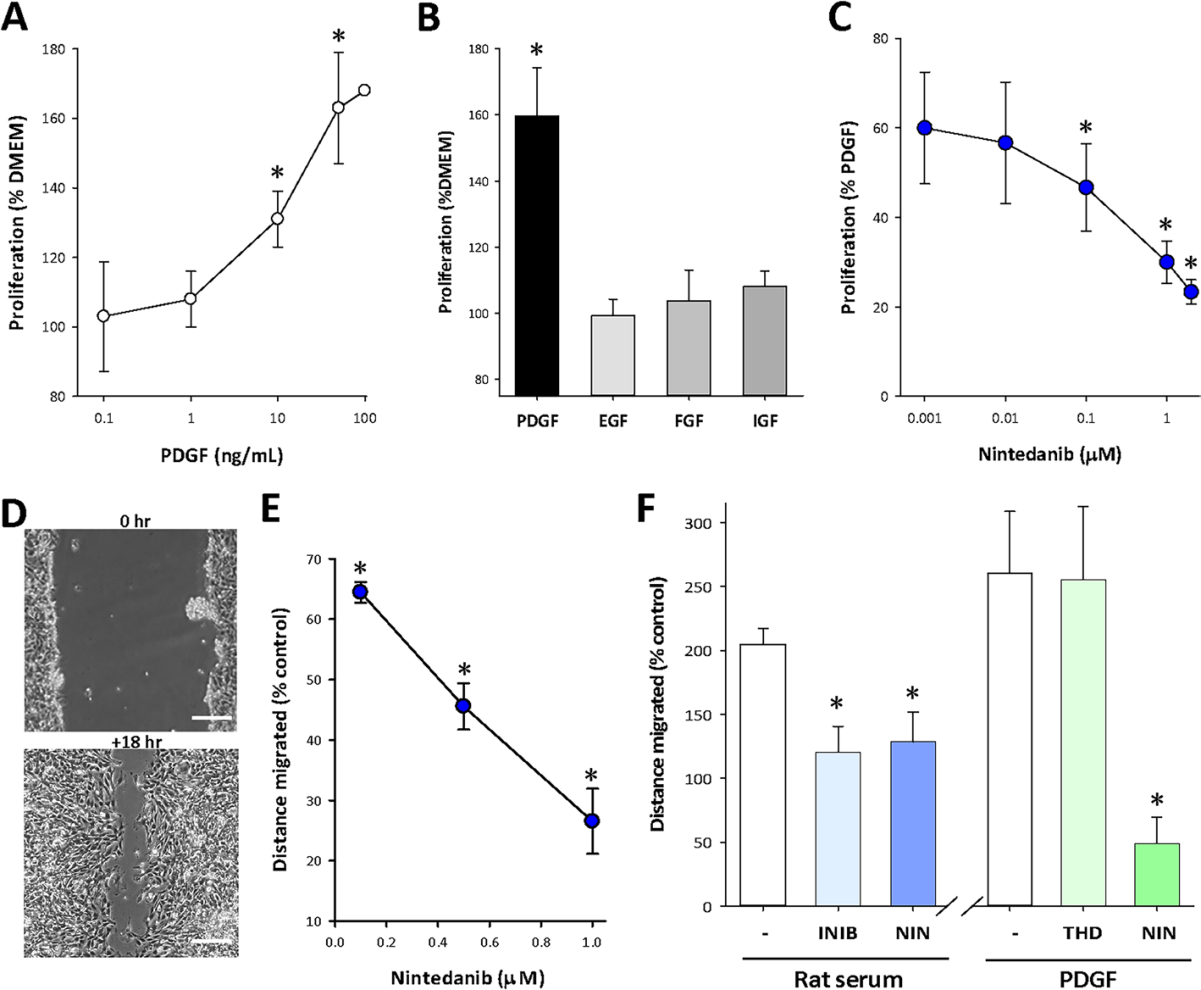
NIN inhibits a PDGF-induced growth and migration of CSMC. (**A**) Concentration-dependent growth of CSMC in response to the mesenchymal growth factor PDGF-BB. *p < 0.05 vs untreated control; n = 5 cell lines in triplicate assay. (**B**) Absence of growth response of CSMC to growth factors EGF, FGF or IGF compared to PDGF-BB (48 h, all 50 ng/mL). *p < 0.05, n = 3 cell lines in triplicate assay. (**C**) Inhibition of PDGF-BB stimulated CSMC growth by nintedanib. *p < 0.05, n = 3 cell lines in triplicate assay. (**D**) Representative images of scrape-wounding assay of cell migration in vitro. Top, appearance after wounding. Bottom, at + 18 h showing cell migration. Scale bar, 125 µm. (**E**) Quantitative analysis of nintedanib-induced inhibition of FCS-stimulated cell migration. *p < 0.05 vs serum control; n = 3 cell lines in triplicate. (**F**) Nintedanib (NIN) blocked PDGDF-induced CSMC migration independently of cell proliferation. Left, inhibition of rat serum-stimulated migration by the PDGF-receptor blocker Imatinib (2 µM) or NIN (1 µM). Right, PDGF-induced CSMC migration was not affected by mitotic inhibition with thymidine (THD, 2 mM) but was blocked by NIN (1 µM). *p < 0.05 vs untreated control; n = 3 cell lines in triplicate assays.

Since earlier studies showed that PDGF-BB was the component of serum responsible for growth stimulation^16,17^, the interaction of NIN with this growth factor was studied further. PDGF-BB (“PDGF”) showed a concentration-dependent stimulation of CSMC proliferation, which was significant for levels > 10 ng/mL and reached a maximum at 50 ng/mL (Fig. 2A), while growth factors such as EGF, FGF and IGF-1 (Peprotech, NJ, USA) were ineffective (50 ng/mL; Fig. 2B). Nintedanib was an effective inhibitor of growth stimulation by PDGF (50 ng/mL), reducing outcomes to control levels at 1 µM (Fig. 2C).

PDGF has multiple actions on smooth muscle cells in other systems, including the stimulation of cell migration^30^. The ability of NIN to inhibit this role in addition to cell proliferation was pursued in CSMC. This involved study of cell migration in response to a cell-clearing linear scrape through confluent cultures (scrape-wounding), with measurement of the mean distance migrated 18 h later (Fig. 2D). Again, Nintedanib blocked cell migration, with 1 µM NIN reducing the distance travelled to about 30% of untreated control (Fig. 2E). To clarify this outcome, we evaluated RS-induced cell migration, comparing the action of NIN to the PDGF-receptor inhibitor Imatinib (Fig. 2F). This showed that NIN (1 µM) and Imatinib (2 µM) were equally effective in preventing CSMC migration. PDGF alone (50 ng/mL) was more effective than 2.5% RS in stimulating migration, an effect that was unchanged in the presence of 2 mM thymidine (THD) to inhibit cell proliferation, but entirely blocked by NIN (1 µM; Fig. 2F). NIN is therefore effective against the separate outcomes of growth factor induced cell proliferation and migration.

**Figure 3:**
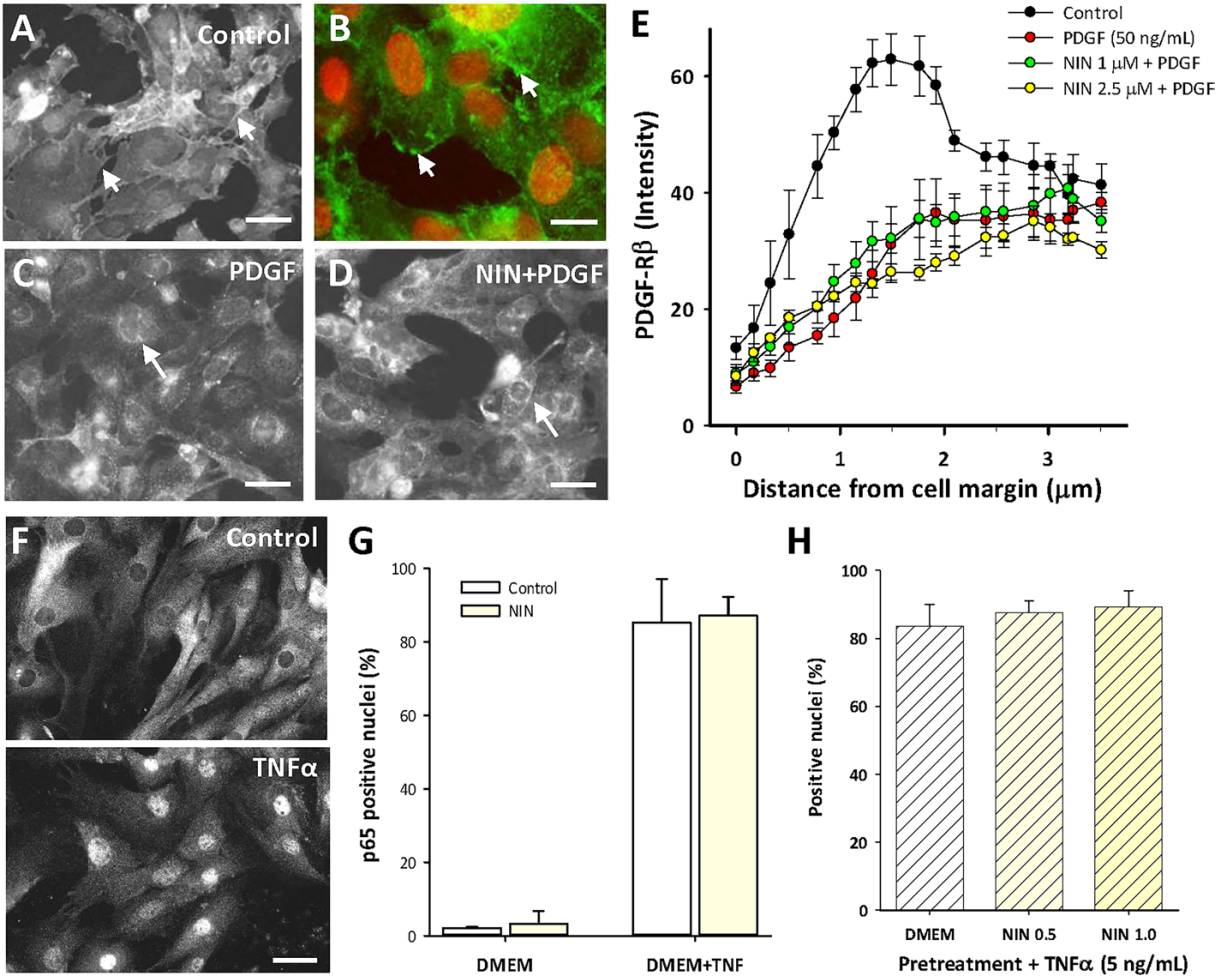
Nintedanib is not an extracellular blocker of the early events of PDGF-Rβ or TNFα-mediated NFκB signaling. (**A**–**D**) Representative images of fluorescence immunocytochemistry for PDGF-Rβ in CSMC. (**A**) Control showing membrane localization of staining that outlined cell edges (e.g., arrows) and was largely absent from the cytosol. (**B**) Higher magnification showing PDGF-Rβ staining (green) of cell membranes (arrows) with red nuclear counterstain. (**C**) PDGF-BB (50 ng/mL, 30 min) decreased membrane staining and induced peri-nuclear staining for PDGF-Rβ (e.g., arrows) that was not affected by nintedanib (**D**; 1 µM). Scale bars, (**A**,**C**,**D**) 50 µm; (**B**) 15 µm. (**E**) Image analysis of staining intensity determined at the cell membranes of CSMC using line scans, showing strong edge-associated labeling in control cells that was lost following exposure to either PDGF or PDGF + nintedanib. Data points at 0.5–2.0 µm from margin were similar for PDGF ± NIN (p > 0.05) and significantly less than control (p < 0.05; n = 3 cell lines, 25 cells per line in 3 replicate experiments. (**F**) Images of immunocytochemistry for p65/NFκB showing control localization in the cytosol (top) and translocation to the nucleus following TNFα (bottom; 50 ng/mL, 30 min). Scale bars, 30 µm. (**G**) image analysis of CSMC nuclei showing that nintedanib (1 µM) did not affect TNFα-induced nuclear p65 localization (30 min; n = 3 cell lines). (**H**) Pretreatment with nintedanib in DMEM (48 h) did not affect subsequent response to TNFα (5 ng/mL; 30 min; n = 3 cell lines).

We next asked whether NIN functioned as an extracellular receptor blocker to limit the actions of PDGF. For this, we used image analysis of CSMC cultures stained for PDGF-Rβ, which is normally localized to the membrane in control CSMC^17^ but is internalized to the cytoplasm upon PDGF stimulation (Fig. 3A–C). A representative image (Fig. 3D) shows this was not affected by pretreatment with NIN (1 µM) and image analysis at high magnification confirmed the membrane-specific high-intensity staining continued to drop with PDGF stimulation in the presence of NIN (Fig. 3E).

This implied that NIN did not directly block receptor activation, at least of PDGF-Rβ and elsewhere, its mechanism of action can include suppression of NFκB signaling^31^. This was pursued through analysis of cytokine-induced activation of the NFκB pathway in CSMC. In control CSMC, there is exclusively cytoplasmic staining for p65/NFκB, which is rapidly converted to uniformly nuclear staining by 30 min after exposure to TNFα (50 ng/mL; Fig. 3F). Quantitation showed that this was unaffected by NIN (20 min pretreatment, 1 µM) in control and TNFα-stimulated conditions (Fig. 3G). To test the possibility that either lower levels of cytokine or longer exposure to NIN might affect this outcome, CSMC lines were exposed to NIN for 48 h before addition of 5 ng/mL TNFα for 30 min. Figure 3H shows that NIN pretreatment at either 0.5 or 1.0 µM did not affect the subsequent response to a reduced level of TNFα, which remained similar to that with 50 ng/mL.

**Figure 4:**
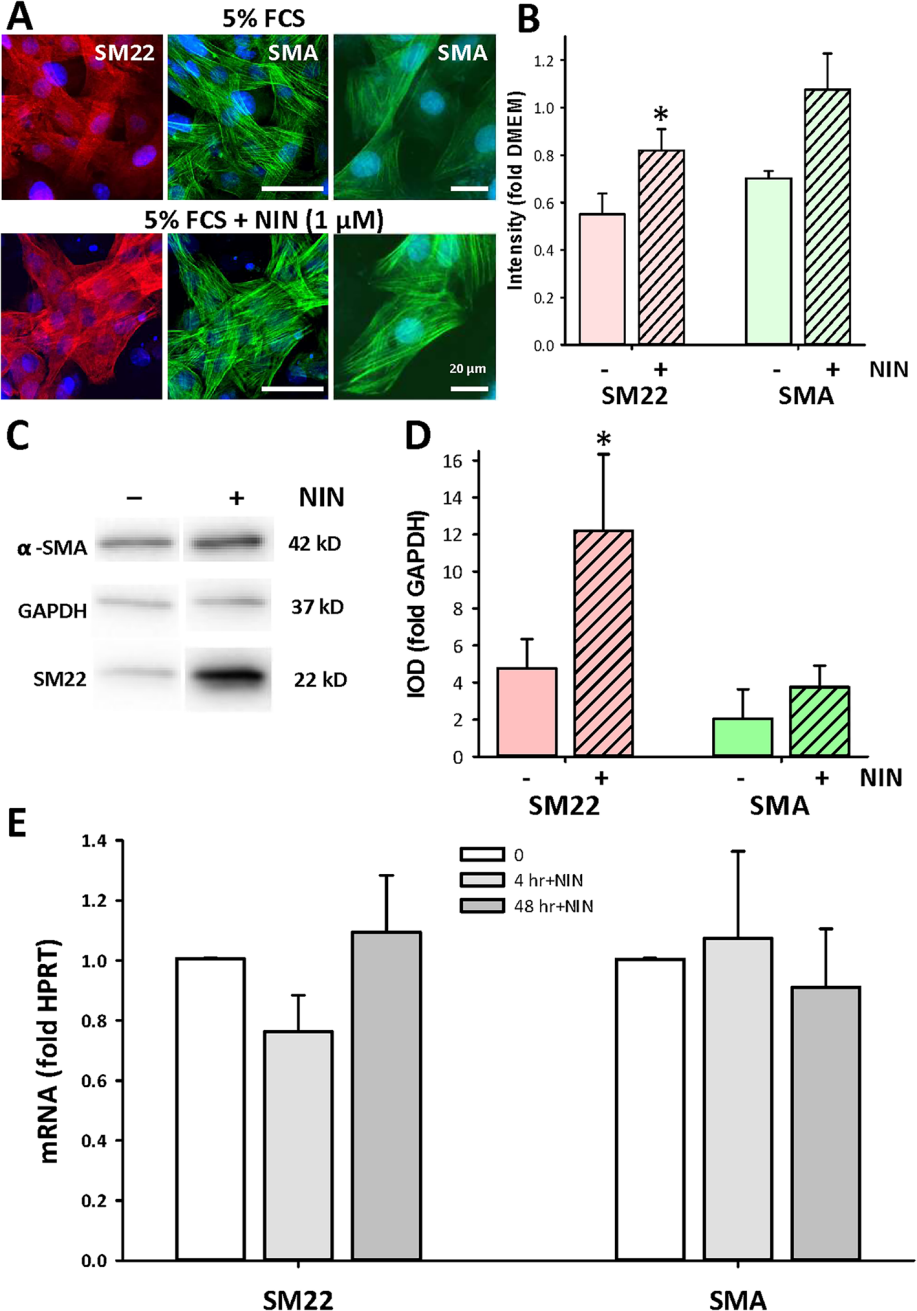
Nintedanib increases expression of smooth muscle marker proteins in high-passage CSMC. (**A**) Immunofluorescent images of high passage CSMC showing labeling for smooth muscle marker proteins SM22 (red) or SMA (green) with nuclei stained with Hoechst (blue). Control (upper) or NIN-treated CSMC (1 µM, 48 h; lower) were imaged identically. Right, higher magnification of SMA staining for each condition. Scale bars, 30 µm (left images) and 20 µm (right images). (**B**) Image analysis of staining intensity for SM22 and SMA in CSMC treated with NIN (1 µM, 48 h). Average line density normalized to black background (n = 4 cell lines, 10–30 cells per cell line, *p < 0.05). (**C**,**D**) Western blotting for SMA and SM22 protein expression in CSMC treated with NIN (1 µM). (**C**) Representative images selected from independent western blots (see Supplementary Fig. 1 for further detail) and (**D**) outcome of image analysis. Equal cell numbers were loaded per lane (n = 5 cell lines, *p < 0.05). IOD, integrated optical density; GAPDH, loading control. *p < 0.05. (**E**) Quantitative PCR analysis of mRNA expression for SM22 and SMA in control vs NIN-treated CSMC (1 µM). All experiments done in triplicates and normalized to HPRT.

The evidence above shows that NIN does not act as a receptor blocker and implies an action involving regulation of gene transcription. This suggested that it might be effective against mitogen-induced dedifferentiation of proliferating CSMC, which was seen earlier^15^ and is well-documented elsewhere^32^. Accordingly, cohort cultures from high passage CSMC lines were maintained in FCS alone or in FCS with 1 µM NIN for 48 h and were studied with immunocytochemistry as well as with qPCR for expression of the smooth muscle markers SM22 or α-smooth muscle actin (SMA). Immunostaining for SM22 and SMA showed significant upregulation of expression of SM22 following NIN exposure compared with control cells, as shown by representative images and quantitative image analysis (Fig. 4A,B). This was separately verified by western blotting, shown in a representative image of the outcomes (Fig. 4C; full images, Supplementary Fig. 1) and in quantitation of replicate experiments (Fig. 4D). However, analysis by qPCR did not show consistent changes in mRNA of either marker when compared to controls, whether early after exposure to NIN (4 h) or by the end of the experiment (48 h; Fig. 4E).

**Figure 5:**
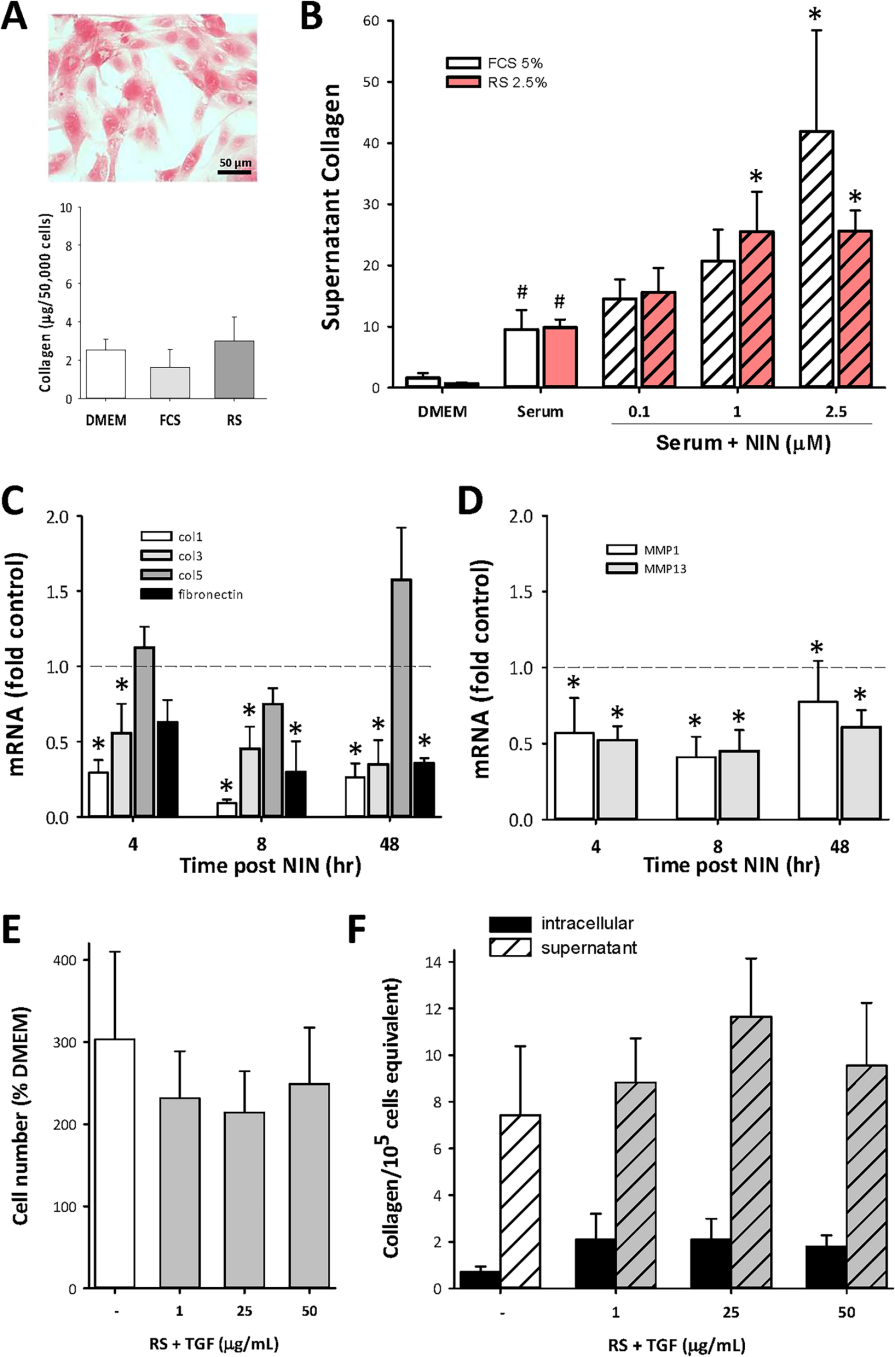
NIN regulates extracellular matrix protein expression in high-passage CSMC. (**A**,**B**) Determination of collagen levels by SirCol quantitative assay. (**A**) Example of histology of Sirius Red staining of serum-stimulated CSMC (top; scale bar, 50 µm) and intracellular collagen levels in CSMC in serum-free (DMEM) or serum-treated conditions. (**B**) Increased supernatant collagen in CSMC cultures with serum or serum + NIN treatment. ^#^p < 0.05 vs DMEM; *p < 0.05 vs serum alone. (**C**,**D**) Quantitative PCR analysis of mRNA for collagen I, III and V, fibronectin (**C**) or metalloproteinases (**D**; MMP1 and MMP13). CSMC were treated with FCS + NIN (1 µM) for 4, 8 or 48 h and data expressed relative to untreated controls. *p < 0.05 vs untreated control. (**E**,**F**) Absence of effect of TGFβ (1–50 ng/mL, 48 h) on CSMC growth (**E**) or total intracellular or secreted collagen (**F**).

Fibrosis is characterized by increased production of extracellular matrix as well as increased cell number, with collagen as the principal component. This was examined in cultured CSMC lines using selective staining with Sirius Red. Figure 5A shows the appearance of Sirius Red staining of CSMC, and the quantitative outcome of measurements of intracellular collagen under serum-free and serum-stimulated conditions. While these outcomes were all similar, extracellular collagen was present in much higher amounts, and was increased by serum stimulation (Fig. 5B). In all cases, direct cell counting was used to normalize data to cell number for assay of intracellular collagen, or for supernatant determinations, to control for varying growth conditions.

Surprisingly, addition of NIN to either FCS- or RS-containing media caused a significant and concentration-dependent increase in supernatant collagen (Fig. 5B), with no measurable changes in intracellular collagen (data not shown). Use of qPCR to determine mRNA levels for the principal isomers of collagen did not show the expected increases with NIN exposure, with decreases in Col1 and Col3, and small increases in Col5 (Fig. 5C). This was matched by decrease in mRNA for fibronectin, suggesting an overall effect of NIN on matrix production at the post-transcriptional level or on collagen stability. The latter is supported by data showing significant decreases in metalloproteinases (MMP) MMP-1 and MMP-13, at both early and later time points (Fig. 5D).

An additional possibility to explain the increased secretion of collagen with NIN treatment was an action through TGFβ (Peprotech), since this is a strongly pro-fibrogenic cytokine, with growth inhibitory actions in low passage CSMC^12^. However, TGFβ failed to affect baseline or RS-induced growth of these high passage CSMC (Fig. 5E) and had no significant effect on either intracellular or secreted collagen (Fig. 5F), consistent with the lack of sensitivity to TGFβ in this cell model as reported earlier^12^.

**Figure 6:**
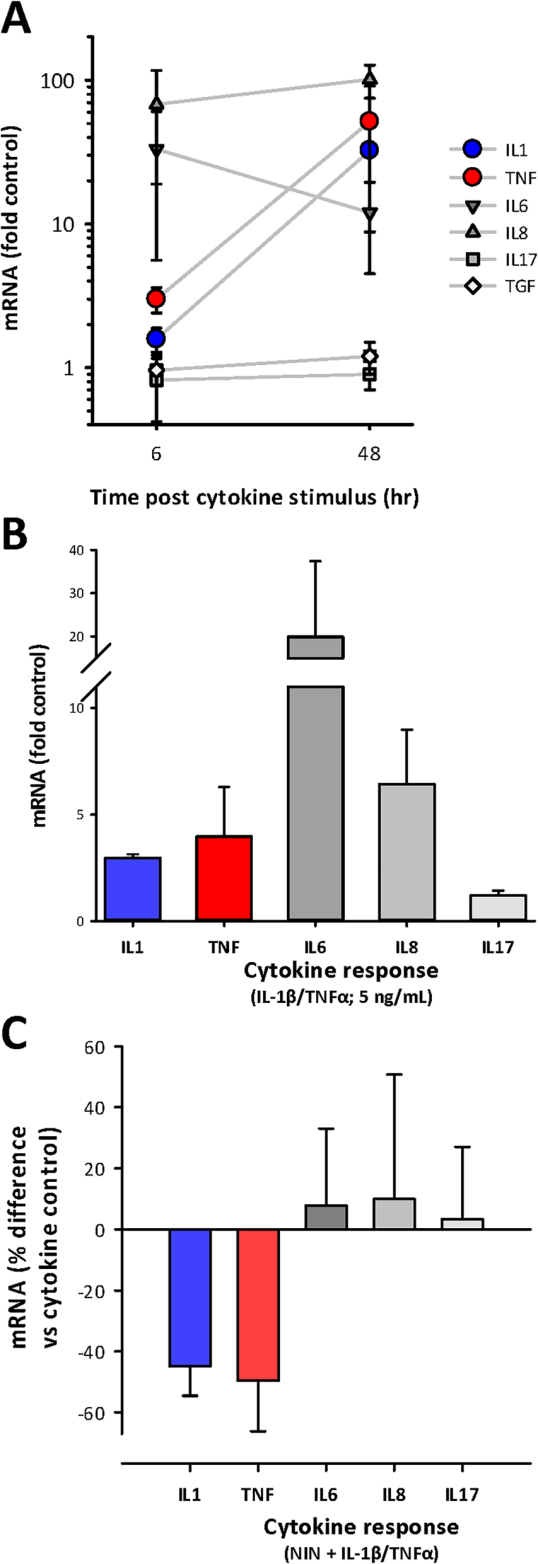
NIN regulates cytokine-induced cytokine expression in CSMC. CSMC were stimulated with the pro-inflammatory cytokines IL-1β and TNFα (50 or 5 ng/mL each) and cytokine expression determined by qPCR at 6 or 48 h. (**A**) Increased expression of the pro-inflammatory cytokines IL-1β, TNFα, IL6 and IL8 but not IL17 or TGFβ at 6 and 48 h. Note large further increases in IL-1β and TNFα between 6 and 48 h. (**B**) Reduced level of stimulation IL-1β/TNFα (5 ng/mL each; 6 h exposure) maintains significant stimulation of IL-1β, TNFα, IL6 and IL8 but not IL17. (**C**) NIN (1 µM) reduces cytokine-induced expression of IL-1β and TNFα, but does not affect IL6, IL8 or IL17. Data expressed relative to cytokine-stimulated control (n = 4 cell lines), with stimulus of TNFα and IL-1β at 5 ng/mL for 6 h. Data, all p < 0.05 vs respective controls.

The potential for NIN to further influence the contribution of intestinal smooth muscle to the inflammatory process was further examined by study of cytokine-induced cytokine expression. This is a potentially important mechanism whereby smooth muscle could actively participate in inflammation, as suggested by earlier reports^33^. Here, we used a combined stimulus of TNFα and IL-1β (50 ng/mL each) and used qPCR to examine alterations in expression of pro-inflammatory cytokines and TGFβ. The results showed 2 major classes of response, with very large increases in IL-1β, TNFα, IL6 and IL8, with no alterations in IL17 or TGFβ (Fig. 6A). Notably, both TNFα and IL-1β increased significantly between 6 and 48 h post stimulation, while other cytokine levels remained constant. There was no effect of NIN treatment (data not shown), but the potential for these cytokines to constitute a supra-physiological stimulus led to testing of reduced amounts. Indeed, Fig. 6B shows that 5 ng/mL of each cytokine still caused large responses by 6 h (Fig. 6B), but NIN treatment now caused significant changes, with mRNA for both TNFα and IL-1β being markedly decreased by NIN, while the other cytokines were not significantly affected (Fig. 6C). This offers insight into a novel inflammatory response of CSMC, as well as demonstrating the potential for an additional effect of nintedanib on regulating inflammatory responses.

**Figure 7:**
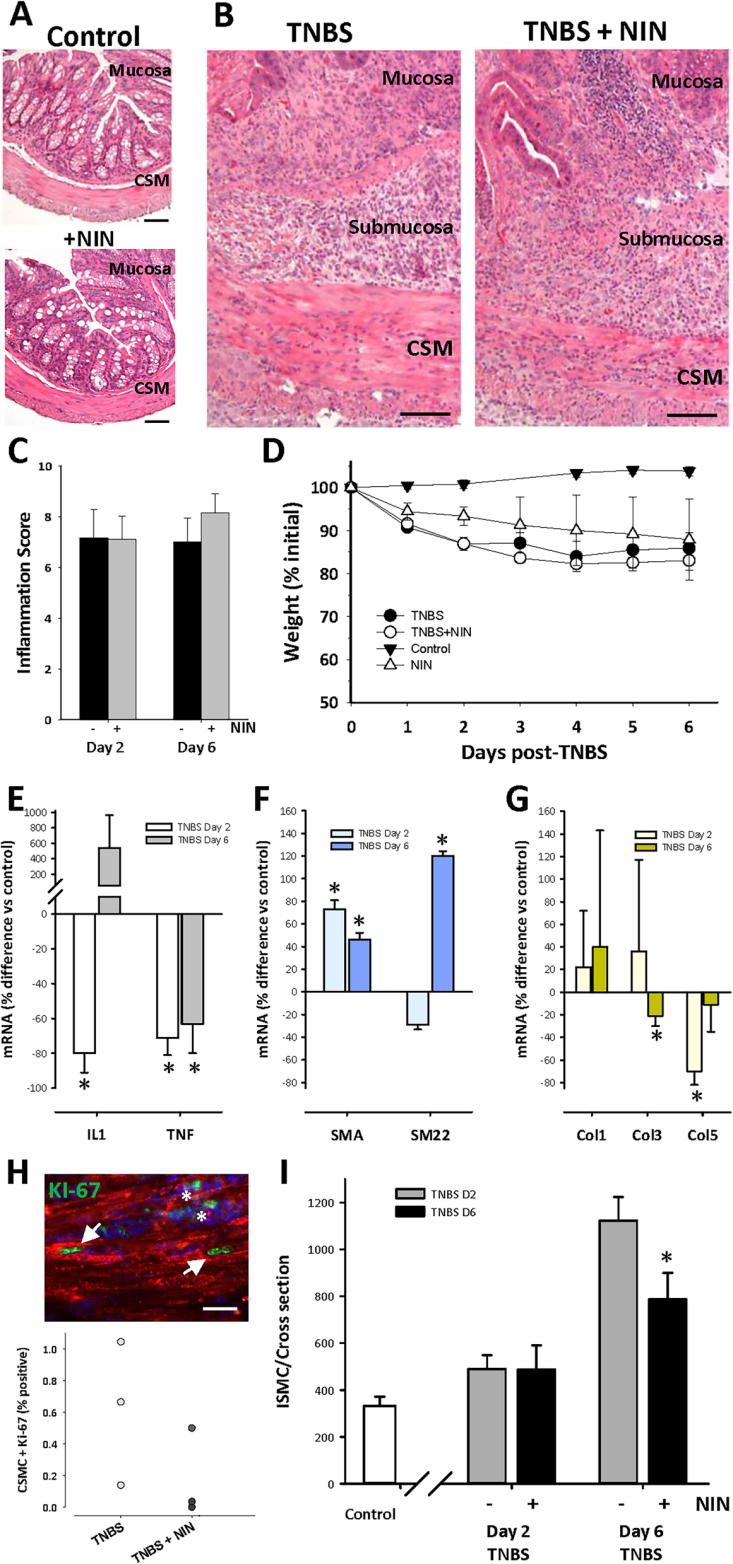
Improved outcomes in TNBS colitis with nintedanib treatment include reduced CSMC growth and increased smooth muscle marker expression. (**A**,**B**) Representative low-power micrographs showing similar histology of mid-descending colon in control mice (top) or control mice with nintedanib (lower; 60 mg/kg, p.o. daily). (**B**) Typical appearance of inflamed colon at Day 6 of TNBS-induced colitis in control TNBS (left) and TNBS + NIN-treated animals (right), showing similar inflammatory damage with diffuse immune infiltrate in the mucosa, submucosa, and smooth muscle layers (CSM, circular smooth muscle). Scale bars: 50 µm (**A**) and 100 µm (**B**). (**C**,**D**) Lack of effect of NIN treatment on mean tissue damage score at Day 2 or 6 of TNBS colitis (**D**), or on animal weights during TNBS colitis (**D**). Note decreasing weight at Days 2 and 6 post-TNBS instillation; p < 0.05 vs control for data > D1. (**E**–**G**) Effect of NIN treatment on mRNA levels in smooth muscle/myenteric plexus tissue at Days 2 and 6 of TNBS colitis. (**E**) Decreased levels of IL-1β and TNFα. (**F**) Increased levels of mRNA of marker proteins SMA and SM22. (**G**) No consistent effect on NIN on collagen expression. Data expressed relative to time matched control cohort animals; n = 4–5/condition per time point. *p < 0.05 vs control. (**H**) Fluorescence image showing growth of CSMC by D2 of TNBS colitis (top), using dual label immunocytochemistry for KI-67 (green) and smooth muscle actin (red) to correlate KI-67 expression with smooth muscle phenotype (arrows) vs immune cells (asterisks). Lower, quantification of KI-67 positive CSMC in Day 2 and Day 2 + NIN mice (n = 3). (**I**) inhibition of CSMC hyperplasia by NIN during TNBS colitis, with significant reduction of typical threefold increase in CSMC number/cross section by Day 6 (*p < 0.05 vs time-matched colitis control).

We next carried out a pilot in vivo study, looking at the effects of NIN on TNBS-induced colitis in mice, an economical approach to work in vivo that duplicates the key parameters seen in rats^10^. Using this model, we have described smooth muscle growth in both rats and mice, as well as increased collagen deposition and altered immunological responses associated with subsequent stricture formation in rats^10,12,34^. This chemically induced inflammation causes a severe transmural colitis centred on the mid-descending colon, that is initially acute (ca. 1–2 days post TNBS) and subsequently displays a chronic phase (Days 4–8) with eventual resolution. We first used histology to determine the effect of NIN (60 mg/kg bid) on control animals, finding no detectable alteration of the epithelial mucosa or underlying smooth muscle layers (Fig. 7A). This dose and its twice daily administration are among the higher amounts used elsewhere in treatment of experimental conditions in mice (e.g. Refs.^35–38^). The appearance of the inflamed colon from NIN + TNBS-treated animals was similar to that of TNBS-treated animals, with both showing mucosal damage and an intense submucosal inflammatory infiltrate that extended into the circular and longitudinal smooth muscle layers (Fig. 7B).

A disease activity index incorporating multiple factors showed no differences between control and NIN-treated animals at either Day 2 or 6 of TNBS (Fig. 7C), and the weight loss expected to occur in this colitis model was similar between control and NIN-treated animals (Fig. 7D). Despite this similarity of macroscopic indicators, qPCR of circular smooth muscle tissue for the inflammatory factors studied above (Fig. 7E) showed that NIN caused significant decreases in mRNA for IL-1β and TNFα on Day 2 post TNBS, with persistent reduction of TNFα levels by Day 6. No significant differences were found in the other markers (not shown).

The similarity of these changes in cytokine levels with those in vitro led us to examine the expression of mRNA for smooth markers SMA and SM22, as well as the collagen isoforms. Figure 7F shows that NIN treatment was associated with approximately a twofold increase in SMA on Days 2 and 6, while the smooth muscle specific marker SM22 was elevated similarly by Day 6, interpreted as evidence for increased phenotype through retention of marker mRNA or more extensive re-differentiation. Outcomes of qPCR for the collagen isoforms yielded highly variable data, with no consistent trends (Fig. 7G).

A prominent action of NIN was inhibition of CSMC proliferation in vitro, and so the presence of mitotic CSMC and the total number of CSMC was determined in vivo. At Day 2 post TNBS, dual-label immunocytochemistry detected positive nuclear labeling with KI-67 in SMA-stained cells (Fig. 7H) in both TNBS- and NIN + TNBS-treated animals. As expected, total CSMC number was slightly increased by Day 2 and showed a nearly threefold increase by Day 6 (Fig. 7I). Notably, NIN treatment did not affect CMSC number by Day 2 but caused a significant decrease in total CSMC by Day 6 (Fig. 7I). Overall, this suggests that NIN preserves smooth muscle cell phenotype through limiting inflammation-induced hyperplasia.

**Figure 8:**
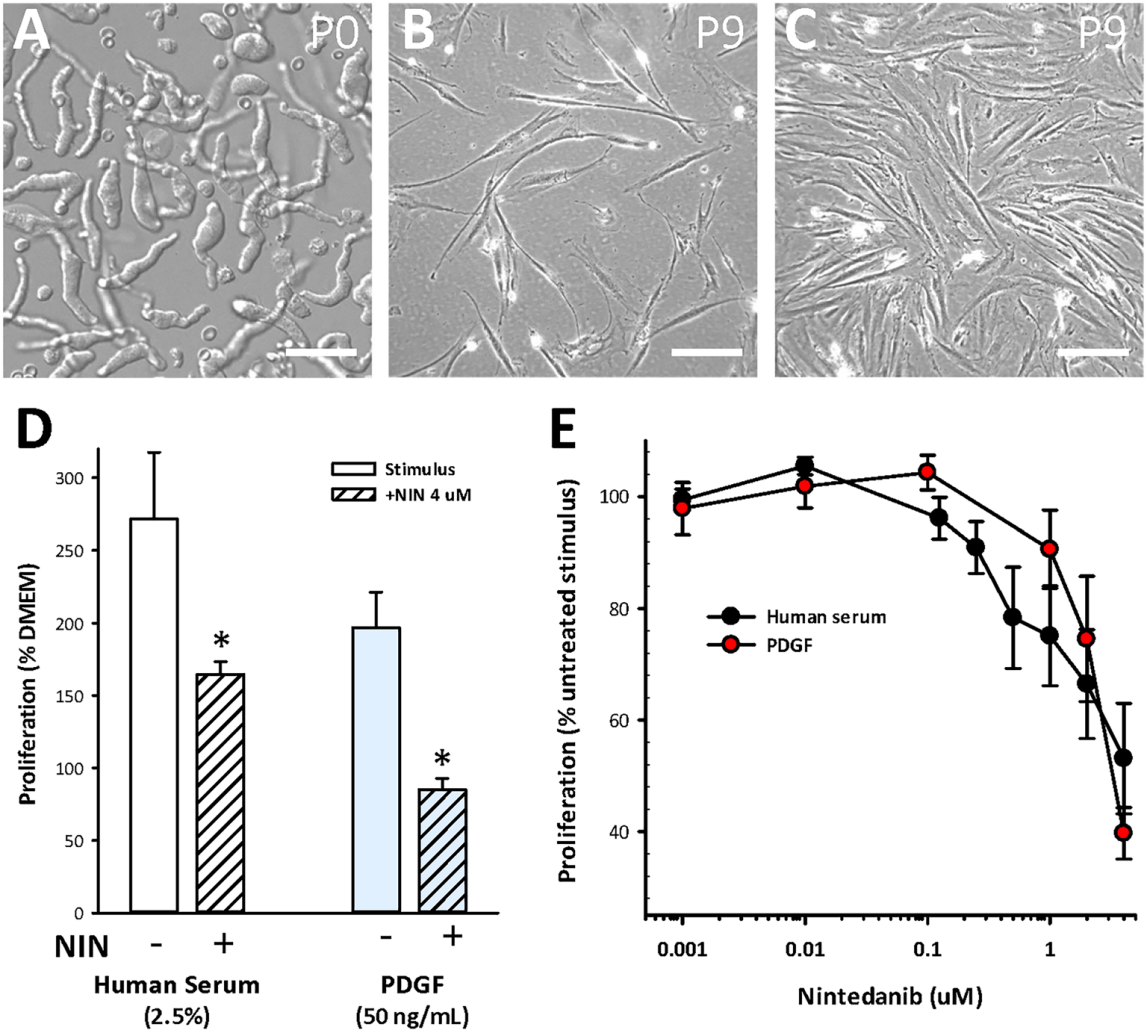
NIN is effective in inhibiting growth of human intestinal smooth muscle cells in vitro. Human intestinal circular smooth muscle cells were obtained from the terminal ileum and maintained to passages 9–12 for assay of growth. Each cell line originates from a different subject. (**A**) Nomarski microscopy image showing appearance of freshly isolated human CSMC. (**B**,**C**) Phase contrast images of low density and confluent cultures at P9, showing elongated bipolar spindle shapes. Scale bars, (**A**) 40 µm; (**B**,**C**) 200 µm. (**D**) NIN (4 µM, 48 h) suppressed proliferation of human CSMC in response to either adult human serum or PDGF-BB (n = 5 cell lines in triplicates). (**E**) Similar concentration–response relationships of inhibition of human serum or PDGF-BB included growth by NIN (n = 3 cell lines). Data, p < 0.05 vs stimulus alone for human serum + NIN ≥ 0.5 µM; PDGF + NIN ≥ 2 µM.

Evidence for a beneficial outcome in vivo in a mouse model of Crohn’s disease suggests the application of NIN to human IBD. In support, key experiments in vitro were repeated using human CSMC derived from normal ileum and maintained as separate cell lines. Figure 8A–C depicts their characteristic appearance upon isolation and during growth at passage 9. In parallel assays to those described above, these cell lines grew and showed a > twofold increase in cell number by 48 h in response to either normal adult human serum or to PDGF in serum-free medium (50 ng/mL), which was significantly inhibited by NIN (4 µM) in each case (Fig. 8D). Comparison of the concentration–response relationships between NIN and HS, or NIN and PDGF showed similar outcomes, with the higher levels of NIN limiting the response to yield CSMC numbers similar to untreated, serum-free controls, without cytotoxicity (Fig. 8E). This further supports the potential application of nintedanib in human IBD, with the intent to specifically target hyperplasia and phenotypic modulation in smooth muscle cells.

## Discussion

Inflammation creates a thickened intestinal wall that compromises neuromuscular regulation of organ motility. The increased number of smooth muscle cells requires innervation for coordinated function, but inflammation also causes axonal damage and even neuronal death that can prevent this. The nature of the hyperplastic smooth muscle cells is affected as well, due to decreased expression of contractile proteins (e.g., Refs.^15,39^). There is also decreased expression of GDNF, the key neurotrophin that supports neural plasticity in the intestine. Overall, this led us to examine opportunities for pharmacological control of smooth muscle hyperplasia. Here, initial evidence shows that NIN controls smooth muscle cell hyperplasia and regulates phenotype. This supports further study in IBD, where it could improve outcomes by promoting homeostasis and regenerative neuroplasticity, while limiting the progression to stricture formation.

The fibrotic pulmonary disease of IPF resembles IBD, with its progressive inflammation and a prominent mesenchymal component. While IPF is otherwise intractable to therapy, both NIN and pirfenidone are beneficial, suggested to reflect their suppression of multiple tyrosine kinase-related pathways that are involved in disease progression. To assess their potential for a role in IBD, and specifically stricture formation in CD, we investigated their effect on intestinal smooth muscle cells, with both in vitro and in vivo approaches. This focused on NIN, since initial work showed it to be a sensitive and effective inhibitor of growth while pirfenidone required far greater amounts to achieve effect. The pharmacodynamics of pirfenidone in IPF may require further study to model this effectively in vitro.

Independent cell lines of adult rat colonic smooth muscle were used, which were described earlier to show epigenetic changes that were also present in the inflamed human intestine in vivo^15^. We concluded that NIN caused the selective suppression of proliferation and the promotion of a differentiated phenotype in vitro, as well as regulation of cytokine expression. A pilot study using the well-studied TNBS model of colitis was successful in showing the suppression of smooth muscle hyperplasia, although without improvement in overall clinical outcomes such as mucosal integrity or disease activity. This is an important extrapolation to events in vivo, since the complexity of chronic inflammation may not be easily studied in preclinical models. For example, the inhibition of signaling by PDGF limited pulmonary fibrosis in experimental models^40^, but treatment of human IPF with the PDGF-Rβ blocker Imatinib was ineffective^41^.

PDGF-BB is the key mitogen for intestinal smooth muscle cells and is present in adult serum, although PDGF can drive proliferation in serum-free media as shown here and earlier^16,17^. While NIN inhibited PDGF-induced growth of smooth muscle cells, this did not affect the early steps of receptor mobilization, as expected for a proposed inhibitor of tyrosine kinases^42^. Similarly, NIN did not inhibit mobilization of p65 to the nucleus following stimulation with TNFα, showing that receptor activation and at least this downstream pathway were unaffected by NIN, and suggesting that its impact may lie in inhibition of transcriptional activation, which is well described for PDGF and the NFκB pathways^43,44^.

Indirect support comes from the finding that NIN also suppressed PDGF-induced CSMC migration independently of a role in cell proliferation. The impact of this phenomenon is unclear, with little research in the intestine beyond an initial report of involvement in the formation of adhesions^45^. In atherosclerosis, the chemotactic movement of vascular smooth muscle cells towards the vascular lumen contributes to formation of the neointima and vascular stenosis^30^. It is probable that both hyperplasia and migration of smooth muscle cells contribute to the locally severe outcome of stricture formation in CD, and further, that NIN appears able to influence multiple aspects of this cellular pathophysiology.

Increased ECM occurs in stricture formation in both an animal model and human IBD, although contributing to a relatively minor extent when compared to the major changes in the cellular compartment. There may also be alterations in mechanical parameters such as compliance and elasticity, due to changes in the molecular profile of ECM^8,12^. NIN consistently caused large increases in secreted collagen in vitro with an altered profile of expression, while expression of the MMPs principally involved in collagen breakdown were decreased. This is an additional aspect of the action of NIN, which may reflect promotion of the outcomes seen during resolution of inflammation, similarly to the growth-inhibiting, profibrotic effects of TGFβ that manifest in the later stages of inflammation. Indeed, smooth muscle cells in the culture model studied here are further characterized by a loss of sensitivity to TGFβ, normally a potent anti-mitotic cytokine^12^. NIN may thus compensate for a critical failure in growth control and inappropriate ECM expression. However, it remains to be seen whether this applies to either the treatment or prevention of stricture formation by NIN in human CD.

The finding that inflammatory stimuli cause high passage CSMC to express multiple pro-inflammatory cytokines is an important addition to the understanding of their responses in vivo. While the concept was established earlier^33,46^, we now show the high potential for hyperplastic CSMC to drive inflammation, with a positive feedback response that creates a nidus of chronic inflammation that might contribute to the spontaneous exacerbation of IBD. That NIN can inhibit cytokine expression speaks to the multi-modal actions of NIN, with outcomes extending well beyond simple inhibition of growth, and deserves further study to increase our understanding and control of the complex events of IBD.

A pilot study of the potential of NIN to influence TNBS-induced colitis in the mouse was successful at several levels, despite limited animal numbers. We found that oral delivery of NIN had no detectable adverse outcomes in either control or colitis conditions, and the early events of acute inflammation were unaffected, interpreted to show the absence of a general anti-inflammatory effect of NIN. This may align with outcomes in treatment of human IPF, where anti-inflammatory approaches are ineffective, but nonetheless, NIN slows disease progression^47^, apparently by limiting fibrotic encroachment upon the alveolar spaces. In line with earlier experiments showing that smooth muscle proliferation becomes significant by Day 2 post-TNBS^11^, the outcomes here showed that NIN suppressed the subsequent CSMC hyperplasia as revealed by the decrease in CSMC number by Day 6. Since this is a key step in stricture evolution, further exploration of a role for NIN in stricture prevention is needed, both directly on mesenchymal hyperplasia, as well as on the local dysregulation of the immune response that correlates with the stricture outcome^12^.

NIN also controlled proliferative responses in high passage human smooth muscle cell lines that parallel the CD phenotype, extending the impact of our findings to potential human therapy. In testing on human smooth muscle cell lines, NIN exerted similar sensitivity and concentration-dependent effects on PDGF or adult human serum, and thus appears to have a similar mechanism of action as in the rat, which further supports early study in human disease.

In summary, the complex actions of NIN in rat intestinal smooth muscle cells include effects on growth control, cell migration and phenotypic modulation, as well as regulation of ECM production and the expression of pro-inflammatory cytokines. This aligns with the current understanding of basic and clinical outcomes in the human disease of IPF. However, important cellular mechanisms are demonstrated that establish NIN as a potentially selective pharmacological tool for study in CD and CD-related stricture formation.

## Supplementary Information

Supplementary Information.

## Data Availability

All data generated or analyzed during this study are represented in this manuscript and its Supplementary Information file. Further information about these or the methods employed to obtain them will be provided by contacting the corresponding author.
