# Supplementary material for: Nintedanib regulates intestinal smooth muscle hyperplasia and phenotype in vitro and in TNBS colitis in vivo
Source: Sci Rep. 2022 Jun 17;12:10275. doi: 10.1038/s41598-022-14491-5 (PMC9206006; doi:10.1038/s41598-022-14491-5)

Supplemental Information - Supplemental Fig 1

Nintedanib regulates intestinal smooth muscle hyperplasia and phenotype in vitro and in TNBS colitis in vivo

Jay Kataria, Jack Kerr, Sandra R Lourenssen and Michael G Blennerhassett*
Gastrointestinal Diseases Research Unit and Queen's University
Kingston, ON K7L 2V7
Canada

Running title: Nintedanib regulates intestinal smooth muscle phenotype

*Correspondence:
M.G. Blennerhassett
GIDRU Wing, Kingston General Hospital
Dept of Medicine, Queen's University
Kingston, ON K7L2V7
Canada
(613) 549 6666 x6523
ORCID: 0000-0002-7195-9417
blennerm@queensu.ca

Supplemental Figure 1.
Entire image of western blot outcomes as displayed in Fig 4C. For each experiment, replicates of cell homogenates were loaded at equal cell numbers per lane into 2 parallel gels. Since the MW of SMA and GAPDH are similar, detection and comparison of SMA and SM22 via the loading control of GAPDH required parallel blots (Blots 1 & 2 as displayed below). After protein separation and transfer, intact membranes were probed with mouse anti-SMA and rabbit anti-SM22 antibodies overnight, and then exposed to anti-HRP-labeled secondary antibodies. This visualized SMA and SM22, and outcomes were imaged before re-exposure to mouse anti-GAPDH antibodies and re-imaging. MW markers were visualized with white light and superimposed on the images (eg, Blot 1 below).

Image analysis was carried out on SMA (Blot 1) and on SM22 and GAPDH (Blot 2). Membrane edges are indicated (blue arrows). The lanes represented in Fig 4C are outlined.

Treatments: -, control; +, NIN 1 µM. MW, molecular weight markers; SMA, α-smooth muscle actin; SM22, smooth muscle-specific protein 22α; GAPDH (loading control), glyceraldehyde 3-phosphate dehydrogenase.

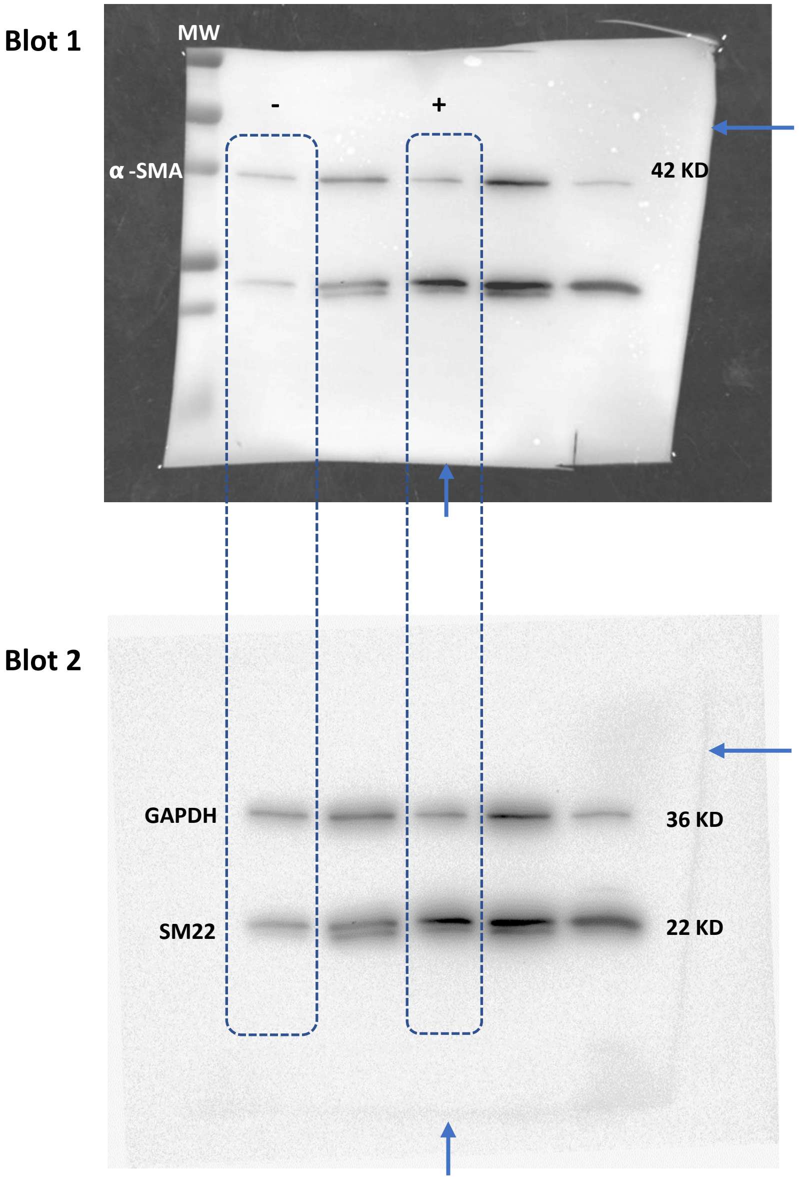

Supplement: Supplementary file 1 — Supplementary Information. [file 41598_2022_14491_MOESM1_ESM.pdf]
